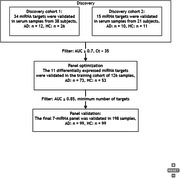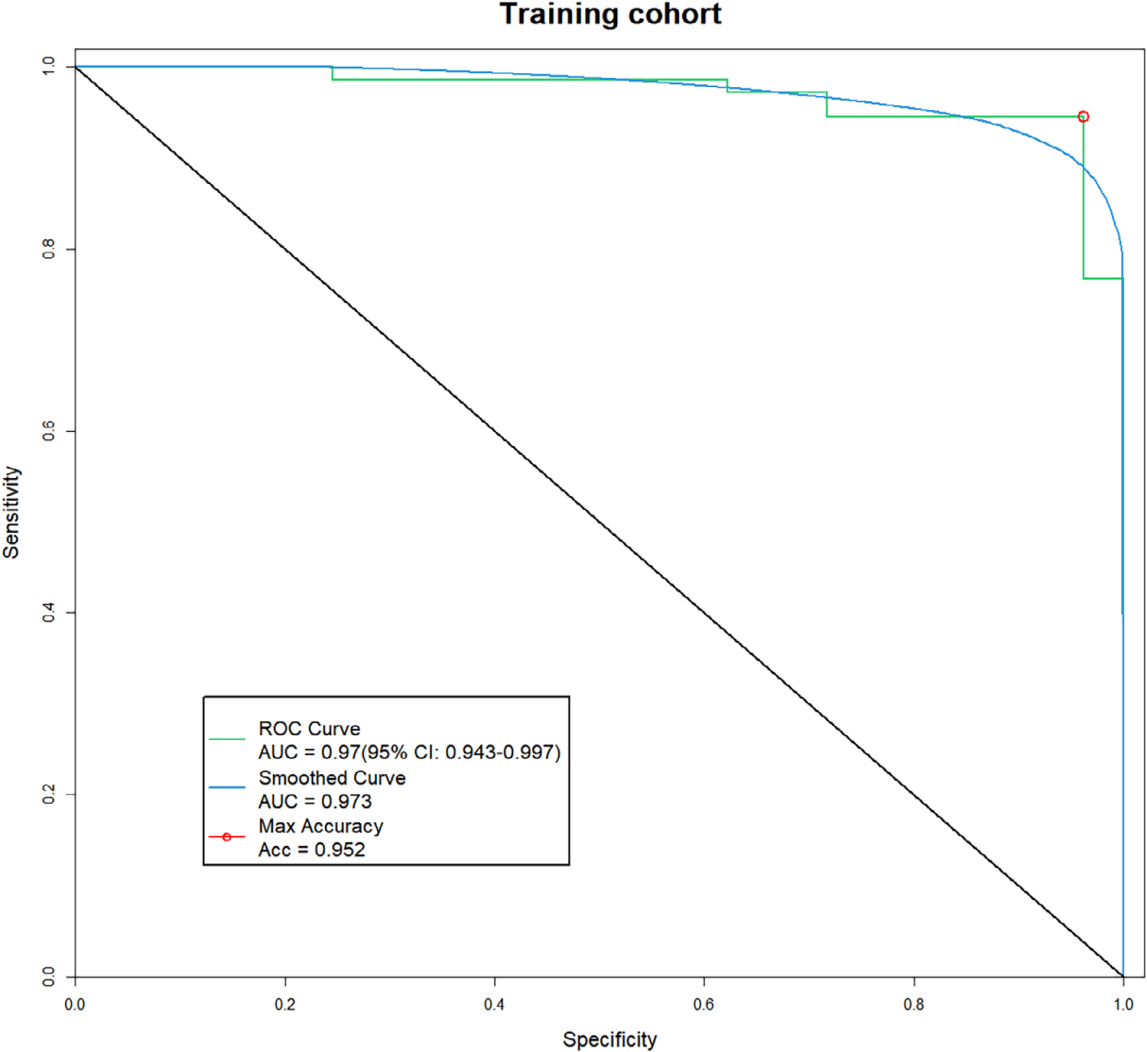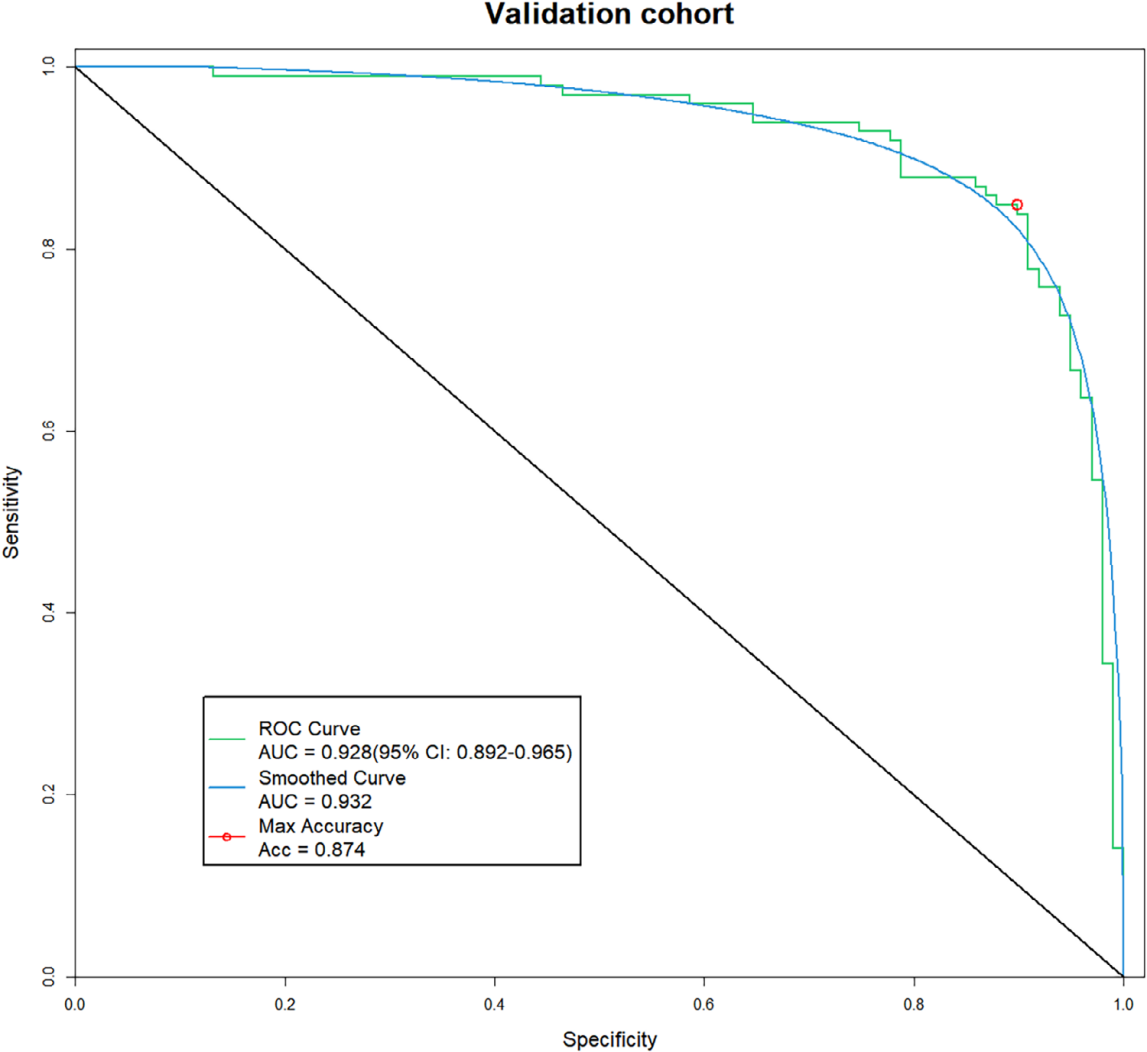# Development of a serum miRNA panel for detection of Alzheimer's Disease

**DOI:** 10.1002/alz.084560

**Published:** 2025-01-09

**Authors:** Xinyu Zhang, Chang Su, Yuan Cao, Songtao Yang, Qi Qin, Yi Tang

**Affiliations:** ^1^ Miracle Biotechnology Inc., Houston, TX USA; ^2^ Department of Neurology & Innovation Center for Neurological Disorders, Xuanwu Hospital, Capital Medical University, National Center for Neurological Disorders, Beijing, Beijing China; ^3^ Xuanwu Hospital Capital Medical University, Beijing, Beijing China

## Abstract

**Background:**

An urgent need exists for minimally invasive testing for accurate detection of Alzheimer’s disease (AD). Circulating microRNAs (miRNAs) have been investigated as a promising candidate biomarker for AD diagnosis and prediction because of their involvement in multiple brain signaling pathways in both health and disease. This study developed and validated a serum miRNA panel in discriminating clinically diagnosed AD from age‐matched cognitively healthy controls.

**Method:**

383 serum samples (194 AD, 189 cognitively healthy controls) were divided into three cohorts: discovery (n=59), training (n=126), and validation (n=198). In the discovery cohort, 49 miRNAs curated from literature databases were verified using individual serum sample via reserve transcriptase‐quantitative Polymerase chain amplification (RT‐qPCR). A logistic regression model was built with 11 differentially expressed miRNAs using the training cohort, and the final panel comprising 7 miRNAs with superior diagnostic performance was established. The diagnostic efficacy of the 7‐miRNA panel was further evaluated in the validation cohort by the receiver operating characteristic (ROC) analysis.

**Result:**

Of the initial 49 screened serum miRNAs, 11 differentially expressed miRNAs were selected for logistic regression model construction based on their potential for detecting AD patients (AUC ≥ 0.7). After model optimization and validation via RT‐qPCR, a 7‐miRNA panel (miR‐146a‐5p, let‐7i‐5p, miR‐21‐5p, miR‐29c‐3p, miR‐92a‐3p, let‐7f‐5p, and miR‐1285‐5p) was identified with area under the curve (AUC) of 0.970 and 0.932 in the training and validation cohorts, respectively. The sensitivity of 7‐miR test was 88%, and the specificity was 85% in the validation cohort.

**Conclusion:**

These findings suggest that the 7‐miRNA signature in serum serves as a novel noninvasive tool for the adjunctive diagnosis of AD. The panel shows promise for clinical application, setting the stage for future studies across diverse populations.